# Attitudes of healthcare workers towards COVID-19 vaccination: a survey in France and French-speaking parts of Belgium and Canada, 2020

**DOI:** 10.2807/1560-7917.ES.2021.26.3.2002047

**Published:** 2021-01-21

**Authors:** Pierre Verger, Dimitri Scronias, Nicolas Dauby, Kodzo Awoenam Adedzi, Cathy Gobert, Maxime Bergeat, Arnaud Gagneur, Eve Dubé

**Affiliations:** 1 ORS PACA (Southeastern Health Regional Observatory), Faculty of Medicine, Marseille, France; 2 Centre d’investigation clinique de l’Hôpital Cochin-Pasteur (CIC 1417), Assistance Publique des Hôpitaux de Paris, Paris, France; 3 Department of Infectious Diseases, CHU Saint-Pierre - Université Libre de Bruxelles (ULB), Brussels, Belgium; 4 Centre for Environmental Health and Occupational Health, School of Public Health, Université Libre de Bruxelles (ULB), Brussels, Belgium; 5 Institute for Medical Immunology, Université Libre de Bruxelles (ULB), Brussels, Belgium; 6 Centre de recherche du CHU de Québec – Université Laval, D’Estimauville, Quebec City, Canada; 7 General Practice, Université Libre de Bruxelles (ULB), Brussels, Belgium; 8 Maxime Bergeat (MS) – French Ministry for Solidarity and Health – Statistical Service (DREES), Paris, France; 9 Department of Pediatrics, centre de recherche du CHUS, Sherbrooke, Quebec, Canada; 10 Université de Sherbrooke-campus de la santé, Sherbrooke, Quebec, Canada; 11 Anthropology Department, Laval University, Quebec City, Canada

**Keywords:** COVID-19, healthcare workers, vaccine acceptance, vaccine safety and efficacy, vaccination campaigns

## Abstract

In October and November 2020, we conducted a survey of 2,678 healthcare workers (HCWs) involved in general population immunisation in France, French-speaking Belgium and Quebec, Canada to assess acceptance of future COVID-19 vaccines (i.e. willingness to receive or recommend these) and its determinants. Of the HCWs, 48.6% (n = 1,302) showed high acceptance, 23.0% (n = 616) moderate acceptance and 28.4% (n = 760) hesitancy/reluctance. Hesitancy was mostly driven by vaccine safety concerns. These must be addressed before/during upcoming vaccination campaigns.

To help control the coronavirus disease (COVID-19) pandemic, unprecedented efforts have been made to develop vaccines against this disease. Since December 2020, several vaccines have been authorised in Canada [1]and the European Union [2,3] for use as early as the end of 2020. In October and November 2020, we conducted a survey of healthcare workers (HCWs) involved in general population immunisation to: (i) measure their willingness to accept future COVID-19 vaccines for themselves and recommend them to their patients; and (ii) explore determinants of acceptance among groups of HCWs according to their views (positive, uncertain, or reluctant towards vaccination).

## A questionnaire-based survey in France as well as in French-speaking parts of Belgium and Canada

We conducted a cross-sectional questionnaire-based survey in October and November 2020 to collect opinions from general practitioners (GPs) in France and French-speaking parts of Belgium (Brussels, Wallonia), as well as nurses in Quebec, Canada. These professionals were chosen because they are involved in general population immunisation. In Quebec, nurses prescribe and administer almost all vaccines, whithout GP supervision. These HCWs are also targeted as a priority group for COVID-19 vaccination [4-7]. In France, the survey took place among a national panel of 2,815 private GPs (i.e. non-salaried employment) set up in 2018, representative of age, gender, region, workload, and HCW density in the GPs' practice zone [8]. Gender was considered in the study, rather than sex, to capture socio-cognitive factors associated with HCWs’ attitudes and practices. In Quebec, we randomly selected 4,000 nurses from the list of the Quebec Order of Nurses and invited those with an available e-mail address (n = 3,973). In Belgium, we invited all GPs practicing in the regions of Brussels and Wallonia (8,412 GPs) through several databases held by different organisations (such as order of GPs or learned societies). Participants were invited to take part online and, in France only, they were contacted by telephone if they had not completed the survey within 4 weeks of invitation to participate. In all, 2,678 HCWs participated: 1,209 of 2,815 GPs (43%) in France, 414 of 8,412 GPs (5%) in French-speaking Belgium, and 1,055 of 3,973 nurses (27%) in Quebec.

The questionnaire, which was pilot-tested for clarity, length, and face validity in the three countries among a separate group of 144 HCWs not included in the survey, concerned ‘future’ COVID-19 vaccines. In this regard, ‘future’ is with reference to the time/context of the study, which occurred when availability or authorisation of any COVID-19 vaccine was not yet a reality. Two main questions were asked to the participants: (i) their willingness to be vaccinated themselves and (ii) their willingness to recommend the vaccines to their patients, using a five-point scale from ‘no, certainly not’ and ‘no, probably not’ to ‘yes, probably’, and ‘yes, certainly’, with a ‘do not know’ option. The other questions are presented in Table 1.

**Table 1:** Data^a^ on attitudes and opinions of healthcare workers^b^ towards future COVID-19 vaccines, in Belgium (Wallonia and Brussels), France (whole country) and Canada (Quebec), October–November 2020 (n = 2,678)

Survey data	COUNTRY Area considered Number of participants (% of total)						p value^d^	Total 2,678 (100%)	
	FRANCE Whole country 1,209^c^ (45%)		BELGIUM Wallonia and Brussels 414^c^ (16%)		CANADA Quebec 1,055^c^ (39%)				
	Number^c^	%^e^	Number^c^	%^e^	Number^c^	%^e^		Number^f^	%^f^
Age in years									
18 to 39	147	12.16	83	19.98	507	48.05	**<0.001**	920	34.37
40 to 59	620	51.27	128	31.05	482	45.68		1,250	46.67
≥ 60	442	36.57	203	48.97	66	6.27		508	18.96
Gender^g^									
Male	736	60.87	237	57.28	121	11.50	**<0.001**	823	30.75
Female	473	39.13	177	42.72	934	88.50		1,855	69.25
If a vaccine against COVID-19 were available, would you be willing to recommend it to your patients?									
Yes, certainly	607	50.19	169	40.73	480	45.48	**<0.001**	1,253	46.77
Yes, probably	352	29.08	167	40.46	360	34.14		879	32.83
No, probably not	63	5.24	5	1.17	20	1.92		80	2.98
No, certainly not	12	0.97	4	0.95	13	1.23		30	1.13
Do not know	175	14.51	69	16.69	182	17.22		436	16.29
If a vaccine against COVID-19 were available, would you be willing to be vaccinated yourself?									
Yes, certainly	562	46.53	164	39.50	452	42.85	0.11	1,175	43.88
Yes, probably	348	28.83	151	36.53	291	27.56		764	28.52
No, probably not	74	6.09	29	7.01	69	6.55		172	6.43
No, certainly not	60	4.93	10	2.36	56	5.28		134	4.99
Do not know	165	13.62	60	14.60	187	17.75		433	16.18
COVID-19 vaccine acceptance									
High	612	50.59	177	42.80	507	48.07	**0.0036**	1,302	48.60
Moderate	295	24.39	138	33.23	224	21.24		616	23.02
Hesitancy/reluctance	302	25.02	99	23.97	324	30.69		760	28.39
Were you vaccinated against seasonal influenza for the winter 2019/20 season?									
Yes	1,031	85.30	347	83.86	636	60.27	**<0.001**	1,876	70.06
No/do not know	178	14.70	67	16.14	419	39.73		802	29.94
Do you sometimes hesitate to recommend some vaccines on the official schedule for your patients, for example, when you have questions about their benefits or risks?									
For adults (≥ 18 years old) with a chronic disease									
Never	893	73.84	318	76.83	505	47.86	**<0.001**	1,561	58.28
Sometimes/often/always/do not know	316	26.16	95	22.98	333	31.54		783	29.23
Does not apply to my practice	0	0.00	1	0.19	217	20.60		334	12.49
Among adults aged ≥ 65 years old									
Never	892	73.82	297	71.66	470	44.56	**<0.001**	1,499	55.97
Sometimes/often/always/do not know	317	26.18	116	27.95	375	35.53		856	31.95
Does not apply to my practice	0	0.00	1	0.39	210	19.91		323	12.08
The safety of a vaccine developed in an emergency, during an epidemic, cannot be considered guaranteed									
Agree^h^	546	45.15	150	36.30	411	38.97	**0.0018**	1,095	40.88
Disagree^h^	513	42.45	198	47.78	444	42.09		1,139	42.55
Do not know	150	12.39	66	15.92	200	18.94		444	16.57
In your opinion, for the population as a whole, how serious is COVID-19 on a scale of 0 to 10?^i^									
Low (scale: 0–4)	332	27.46	NA	NA	NA	NA	NA	332	27.46
Moderate/do not know (scale: 5–6)	376	31.08	NA	NA	NA	NA		376	31.08
High (scale: 7–10)	501	41.46	NA	NA	NA	NA		501	41.46
It is preferable to acquire immunity against infectious diseases naturally (by having the disease) than by vaccination									
Agree^h^/do not know	99	8.22	31	7.45	134	12.67	**0.0062**	291	10.87
Disagree^h^	1,110	91.78	383	92.55	921	87.33		2,387	89.13
I trust science to develop safe effective new vaccines									
Agree^h^	1,156	95.59	390	94.32	971	92.00	**0.0057**	2,500	93.34
Disagree^h^/do not know	53	4.41	24	5.68	84	8.00		178	6.66
I trust the ministry of health to ensure that vaccines are safe									
Agree^h^	918	75.90	294	70.93	918	87.04	**<0.001**	2,205	82.35
Disagree^h^	218	18.07	96	23.28	74	6.98		313	11.67
Do not know	73	6.03	24	5.79	63	5.98		160	5.98

COVID-19: coronavirus disease; GP: general practioner; NA: not applicable.

^a^ Data are weighted, unless otherwise specified.

^b^ In France (whole country) and Belgium (Brussels and Wallonia) GPs took part in the survey. In Canada (Quebec) nurses took part.

^c^ Unweighted figures.

^d^ The differences between the three country samples is assessed using a chi-squared test (with Rao–Scott correction).

^e^ Weighted percentages (for each country, the percentages were weighted according to age, gender and region).

^f^ Weighted for the whole sample (including the three countries).

^g^ Gender was considered in the study, rather than sex, to capture socio-cognitive factors associated with healthcare workers’ attitudes and practices.

^h^ Strongly or somewhat.

^i^ Question asked to participants in France only.

The bold font indicates statistical significance at the p<0.05 level.

A score of presumptive acceptance of future COVID-19 vaccines was constructed based on the responses to the two questions in the survey about COVID-19 vaccines. The score was derived from awarding points per participant, depending on the different possible responses given, with 0 point for an answer stating ‘no certainly not’, one point for ‘no, probably not’, two points for ‘yes, probably’ and three points for ‘yes, certainly’. ‘Do not know’ answers did not get any points and were considered separately. The points obtained for each of the two questions per participant were then summed to obtain the score (Cronbach’s alpha: 0.83; range: 0–6). We then used the score to categorise participants according to their degree of ‘COVID-19 vaccine acceptance’: ‘high acceptance’ (score > 4), ‘moderate acceptance’ (score = 4) and ‘hesitancy or reluctance’ (score < 4 or answers ‘do not know’ to at least one of the two questions). We weighted the samples for age, gender and region. All analyses used two-sided p-values, defined statistical significance as p < 0.05, and were performed with Stata 14, R 4.0.1 and SAS 9.4.

## Attitudes and perceptions of healthcare workers towards COVID-19 vaccines

Among the 2,768 participants, 79.6% (n = 2,132) would certainly or probably recommend a future COVID-19 vaccine to their patients; and 72.4% (n = 1,939) would certainly or probably agree to be vaccinated with it (Table 1). 

Pooling the answers to the two questions about willingness of HCWs to get vaccinated with future COVID-19 vaccines and their intention to recommend the vaccines to their patients into the variable ‘COVID-19 vaccine acceptance’ and using the score yielded 48.6% (95% confidence interval (CI): 46.2–51.0) of participants with high acceptance, 23.0% (95% CI: 21.1–25.1) with moderate acceptance, and 28.4% (95% CI: 26.3–30.6) with hesitancy or reluctance. Moreover, 40.9% of participants reported that the safety of vaccines developed in an emergency during an epidemic cannot be guaranteed (Table 1).

## Concerns about the safety of future COVID-19 vaccines: main drivers of hesitancy or reluctance

A multi-model averaged polytomous logistic regression [9], with high acceptance as the reference, showed that this opinion about the safety of vaccines developed in an emergency was, by far, the most important factor independently associated with hesitancy or reluctance and with moderate acceptance (Table 2): this factor had the highest partial Nagelkerke’s pseudo-R^2^ (17%, Table 3), a statistic computed to measure the contribution of each explicative variable to the dependent variable [10]. The replication of this result in separate analyses for each country (data not shown) suggests that these findings are robust and, above all, transcend background cultural, professional and social factors [11,12]. Moreover, we found minimal but not statistically significant changes in HCWs' attitudes after press releases about a COVID-19 vaccine’s effectiveness and safety [13].

**Table 2:** Identifying certain factors associated with moderate acceptance or hesitancy/reluctance towards COVID-19 vaccines among healthcare workers in France (whole country), Belgium (Wallonia and Brussels) and Canada (Quebec), October–November 2020 (n = 2,423^a^)

Factors	COVID-19 vaccine acceptance^b^ (ref.: high acceptance^c^)			
	Moderate acceptance		Hesitancy/reluctance	
	aOR	95% CI	aOR	95% CI
Healthcare worker characteristics				
Age in years				
18 to 39	Ref.	Ref.	Ref.	Ref.
40 to 59	**0.74**	**0.58–0.94**	**0.57**	**0.43–0.74**
60 and over	**0.74**	**0.41–0.76**	**0.35**	**0.24–0.50**
Gender^d^				
Male	Ref.	Ref.	Ref.	Ref.
Female	1.22	0.96–1.55	**1.89**	**1.44–2.49**
Country and type of healthcare worker				
France (whole country), GPs	Ref.	Ref.	Ref.	Ref.
Belgium (Brussels and Wallonia), GPs	**1.48**	**1.09–2.02**	**1.64**	**1.14–2.35**
Canada (Quebec), nurses	**0.53**	**0.40–0.71**	**0.58**	**0.42–0.80**
Personal vaccination: were you vaccinated against seasonal influenza for the winter 2019/20 season?				
No/non-response	Ref.	Ref.	Ref.	Ref.
Yes	**0.66**	**0.49–0.88**	**0.37**	**0.28–0.50**
Vaccine recommendation: hesitancy to recommend vaccines to at-risk patients				
None	Ref.	Ref.	Ref.	Ref.
Yes	**1.36**	**1.09–1.70**	**1.58**	**1.25–2.01**
Perceived risks of new vaccines in emergencies: the safety of a vaccine developed in an emergency, during an epidemic, cannot be considered guaranteed				
Disagree	Ref.	Ref.	Ref.	Ref.
Agree	**3.01**	**2.38–3.79**	**12.93**	**9.65–17.33**
Do not know	**1.89**	**1.39–2.56**	**5.81**	**4.06–8.31**
Perceived utility of vaccines: it is preferable to acquire immunity against infectious diseases naturally (by having the disease) than by vaccination				
Disagree	Ref.	Ref.	Ref.	Ref.
Agree/do not know/non-response	**1.57**	**1.05–2.36**	**1.74**	**1.15–2.63**
Trust in science and in the ministry of health				
I trust science to develop safe effective new vaccines				
Disagree/do not know/non-response	Ref.	Ref.	Ref.	Ref.
Agree	0.81	0.45–1.46	**0.38**	**0.22–0.65**
I trust the ministry of health to ensure that vaccines are safe				
Disagree	Ref.	Ref.	Ref.	Ref.
Agree	0.82	0.58–1.16	**0.34**	**0.24–0.47**
Do not know/no response	1.10	0.61–1.97	1.25	0.73–2.13
Period of questionnaire completion				
Before 10 November 2020^e^	Ref.	Ref.	Ref.	Ref.
From 10 November 2020 onwards	0.97	0.73–1.29	0.84	0.62–1.15

aOR: adjusted odds ratios; CI: confidence interval; COVID-19: coronavirus disease; GP: general practitioner; Ref.: reference.

^a^ The three country samples were merged. 255 observations were excluded because vaccine recommendation did not apply to some participants’ practices (these exclusions do not significantly change the results).

^b^ The COVID-19 vaccine acceptance variable was based on the results of two questions: (i) willingness to recommend the COVID-19 vaccines to patients, (ii) willingness to get vaccinated against COVID-19. ‘High acceptance’ indicates certitude of willingness to get vaccinated and to recommend vaccinations to patients, ‘moderate acceptance’ indicates the participant’s intention to probably self-vaccinate and recommend the vaccine to patients, and ‘hesitancy/reluctance’ refers to uncertainty (‘do not know’ responses) or reluctance to be self-vaccinated or to recommend COVID-19 vaccines to patients.

^c^ Because the dependent variable (i.e. acceptance) has three categories ((i) ‘high acceptance’, (ii) ‘moderate acceptance’ and (iii) ‘hesitancy/reluctance’), there are two comparisons (multiple polytomous logistic regression): ‘moderate acceptance’ is compared to ‘high acceptance’ (reference), and ‘hesitancy/reluctance’ is also compared to ‘high acceptance’. We found no issue of multicollinearity (variance inflation factor < 5).

^d^ Gender was considered in the study, rather than sex, to capture socio-cognitive factors associated with healthcare workers’ attitudes and practices.

^e^ Date of European media coverage of the press release about a COVID-19 vaccine’s effectiveness and safety [13].

Terms in bold indicate statistical significance at the p < 0.05 level.

**Table 3:** Ranking of factors associated with COVID-19 vaccine acceptance among healthcare workers, using unweighted data, France (whole country), Belgium (Brussels and Wallonia) and Canada (Quebec), October–November 2020

Factors	Global		
	Rank^a^	Importance Weight^b^	Partial R^2c^ (%)
The safety of a vaccine developed in an emergency, during an epidemic, cannot be considered guaranteed	1	1.00	17.0
I trust the ministry of health to ensure that vaccines are safe	2	1.00	4.0
Personal vaccination against seasonal influenza	3	1.00	2.0
I trust science to develope safe effective new vaccines	4	1.00	1.0
Score of hesitancy about recommending vaccines to at-risk patients	5	1.00	1.0
It is preferable to acquire immunity against infectious diseases naturally (by having the disease) than by vaccination	6	0.88	0.0
Period of questionnaire completion^d^	7	0.20	0.0
Total Nagelkerke Pseudo-R^2c^	NA	NA	30.0

COVID-19: coronavirus disease; NA: not applicable.

^a^ The ranking of the explanatory variables according to their importance in the model, in terms of strength of association, can be derived from the values of the importance weights: 0.00 to <0.50, no association; 0.50 to <0.75, weak association; 0.75 to <0.95, positive association; 0.95 to <0.99, strong association; 0.99 to ≤1.00, very strong association.

^b^ Importance of the explanatory variables in the model.

^c^ Part of the dependent variable variance explained by the explanatory variable: the sum of all partial R^2^ gives the Nagelkerke Pseudo-R^2^ (a statistic comparable to R^2^ in linear regressions).

^d^ Before 10 November (date of European media coverage of the Pfizer press release about a COVID-19 vaccine’s effectiveness and safety) and on and after this date [13].

We also found that distrust in the ministry of health to ensure vaccine safety, was the second factor most strongly associated with lower COVID-19 vaccines acceptance (partial R^2^: 4%, Table 3).

Unsurprisingly, history of personal vaccination against seasonal influenza was also independently predictive of HCWs' acceptance of COVID-19 vaccines (Table 2), as previously found in the influenza A(H1N1)pdm09 pandemic [14]; this suggests the weight of personal habits regarding vaccination in HCWs.

## Ethical statement

The ethics boards of the University-Hospital-Centre Saint-Pierre (Belgium, CE/20–10–14), the Conseil national de l’information statistique (France, CNIS, avis n°114/H030) and the University-Hospital Centre of Québec–Laval University (Québec, #2021–5286) approved the study protocol and questionnaire.

## Discussion

For several months now, a number of studies in several countries have indicated negative attitudes towards future vaccines against COVID-19, in proportions of up to or exceeding 30–40% of the general population [15,16]. A principal reason for such attitudes seems to be the concern that the new vaccines will not be safe [16]. Regarding HCWs, past experience with pandemic influenza vaccination suggests that not all of them will agree to be vaccinated against COVID-19 [14]. However, currently, there are only few publications about HCWs’ acceptance to get vaccinated with COVID-19 vaccines and, to our knowledge, none about their intention to recommend these vaccines to their patients. Previous reports suggest that willingness to get vaccinated lies between 60% and 90% among physicians in Greece (February 2020) and France (March–July 2020) [17,18] and between 40% and 60% among nurses in Hong Kong, China (February–March 2020) and France [18,19]. In our study, 72.4% of participating HCWs would be in favour of getting vaccinated with a future COVID-19 vaccine and 79.6% would be willing to recommend it to their patients.

It is often mistakenly believed that HCWs’ attitudes must be positive towards vaccines because they have scientific and medical training. Nevertheless, HCWs are not a homogenous group and most are not experts in the field of vaccination [20]. Immunisation is moreover not an important part of their initial training [21], and professionals attracted by a further education in this field tend to be those already ‘convinced’ about the benefits of vaccines. Numerous studies indicate that vaccine hesitancy exists among HCWs, at prevalence and intensity levels that vary inversely with their level of training on this topic [11,22-24]. In our work, the perception that vaccines developed in an emergency cannot be guaranteed safe, appeared to play an important role on acceptance of COVID-19 vaccines. Analyses restricted to French GPs even suggest that despite reassuring evidence from phase 2 clinical trials [25], the perception of these vaccines' hypothetical risks is more influential than the perception of the harm potentially resulting from the pandemic (ranked fifth, data not shown). Yet, the consequences of the pandemic are widely documented and experienced individually by most GPs in their daily practice.

Our survey also found that distrust in the ministry of health to ensure vaccine safety also seemed to play a role in lower COVID-19 vaccines’ acceptance. Trust in the institutions through which information about vaccines is delivered is an essential driver of vaccine acceptance not only for the general population but also for HCWs, as long as social context shapes how information is interpreted and used [11,22]. This trust has been tested since the pandemic began by a number of controversies (e.g. effectiveness of masks and specific old or new drugs). The minimal changes in HCWs' attitudes after the press releases about a COVID-19 vaccine's effectiveness and safety [13] raises concerns that these attitudes might not be easily amenable to change in some healthcare professionals, especially also given the relatively low trust of HCWs in the pharmaceutical industry [20]. It is essential to regularly monitor the attitudes and practices of healthcare professionals toward COVID-19 vaccines in the period ahead, not only because of their role in vaccination campaigns, but also, of course, because they are involved in patient care.

### Strengths and limitations

Although participation rates in France and Quebec were high for online surveys, generalisation of the results presented here requires caution, and confirmation in other countries is warranted. Reporting biases, especially social desirability biases are possible. These could potentially lead to over-reporting high COVID-19 vaccine acceptance, which would not change our conclusions. Causal inferences cannot be drawn from this cross-sectional and observational study. However, the model averaging approach enabled us to obtain robust estimates and rank the explicative factors.

### Conclusion

Because HCWs should be among the first to receive the vaccines, their concerns about the safety of these vaccines must be addressed as early as possible [26]. Building trust will require that independent committees and trusted bodies (such as national immunisation committees and professional associations) provide HCWs with credible information about these vaccines' safety and efficacy. In this respect, the monitoring of side effects of authorised vaccines and regular and reactive feedback to healthcare professionals are essential to ensure trust in both COVID-19 vaccines and health authorities. We also need a framework, guidelines, approaches and tools that have proven their effectiveness in addressing HCWs' vaccination concerns that may persist. The upcoming vaccination campaign offers an unprecedented opportunity to develop and evaluate effective interventions targeting HCWs to address their general and specific vaccine hesitancy.
